# MerCat2: a versatile *k*-mer counter and diversity estimator for database-independent property analysis obtained from omics data

**DOI:** 10.1093/bioadv/vbae061

**Published:** 2024-04-24

**Authors:** Jose L Figueroa, Andrew Redinbo, Ajay Panyala, Sean Colby, Maren L Friesen, Lisa Tiemann, Richard Allen White

**Affiliations:** Department of Bioinformatics and Genomics, North Carolina Research Center (NCRC), The University of North Carolina at Charlotte, Kannapolis, NC 28081, United States; Department of Bioinformatics and Genomics, Computational Intelligence to Predict Health and Environmental Risks (CIPHER), The University of North Carolina at Charlotte, Charlotte, NC 28223, United States; Department of Bioinformatics and Genomics, North Carolina Research Center (NCRC), The University of North Carolina at Charlotte, Kannapolis, NC 28081, United States; Department of Bioinformatics and Genomics, Computational Intelligence to Predict Health and Environmental Risks (CIPHER), The University of North Carolina at Charlotte, Charlotte, NC 28223, United States; High Performance Computing (HPC) Group, Pacific Northwest National Laboratory, Richland, WA 99352, United States; Biological Sciences Division, Pacific Northwest National Laboratory, Richland, WA 99352, United States; Department of Plant Pathology, Washington State University, Pullman, WA 99163, United States; Department of Plant, Soil and Microbial Sciences, Michigan State University, East Lansing, MI 48824, United States; Department of Bioinformatics and Genomics, North Carolina Research Center (NCRC), The University of North Carolina at Charlotte, Kannapolis, NC 28081, United States; Department of Bioinformatics and Genomics, Computational Intelligence to Predict Health and Environmental Risks (CIPHER), The University of North Carolina at Charlotte, Charlotte, NC 28223, United States

## Abstract

**Motivation:**

MerCat2 (“Mer—Catenate2”) is a versatile, parallel, scalable and modular property software package for robustly analyzing features in omics data. Using massively parallel sequencing raw reads, assembled contigs, and protein sequences from any platform as input, MerCat2 performs *k*-mer counting of any length *k*, resulting in feature abundance counts tables, quality control reports, protein feature metrics, and graphical representation (i.e. principal component analysis (PCA)).

**Results:**

MerCat2 allows for direct analysis of data properties in a database-independent manner that initializes all data, which other profilers and assembly-based methods cannot perform. MerCat2 represents an integrated tool to illuminate omics data within a sample for rapid cross-examination and comparisons.

**Availability and implementation:**

MerCat2 is written in Python and distributed under a BSD-3 license. The source code of MerCat2 is freely available at https://github.com/raw-lab/mercat2. MerCat2 is compatible with Python 3 on Mac OS X and Linux. MerCat2 can also be easily installed using bioconda: mamba create -n mercat2 -c conda-forge -c bioconda mercat2

## 1 Introduction

Massively parallel sequencing (MPS) of whole ecosystems has elucidated the complexity of microbial composition, functions, and potential roles. With the scaling of terabytes of sequencing data, the illumination of genomes is ever more present, resulting in the expansion of reference databases needed for matching data using compositional, functional, and assembly-based methods. Kraken2 (Wood *et al.* 2019), MetaPhlAn2 (Truong *et al.* 2015), DRAM (Shaffer *et al.* 2020), MicrobeAnnotator (Ruiz-Perez *et al.* 2021), and MetaCerberus (Figueroa III *et al.* 2024) provide database-dependent analysis of composition and function directly from metaome data (e.g. metagenomics and/or metatranscriptomics). However, database-dependence approaches fail to utilize, classify, or model all data due to a lack of references within a database. In addition, *de novo* assembly approaches, although recently improved for complex data types (Howe *et al.* 2014, Li *et al.* 2015), cannot assemble all data into contigs, leaving data underutilized. Open-source reference sequence databases are facing several challenges, including finding ongoing funding; many are moving to subscription-based access (e.g. KEGG www.kegg.jp/kegg/) and discontinuation (e.g. CAMERA http://camera.calit2.net/). Therefore, robust tools are needed to analyze these data in a database-independent manner.

Database-independent property analysis (i.e. DIPA), which utilizes counting of *k*-mer subsequences (of length *k*) from sequence reads without a reference sequence database for matching query data. DIPA-based *k*-mer counting provides rapid and robust microbial community analysis and characterization without the biases or limitations of sequence databases (Jiang *et al.* 2012) and/or *de novo* assembly to compare and contrast sequence datasets. *k*-mers are critical to assembly (Li *et al.* 2015), counting (Zhang *et al.* 2014), partitioning (Howe *et al.* 2014), genomic binning (Wu *et al.* 2016), and classification (Jiang *et al.* 2012). *k*-mer-based counting is among the fastest approaches for profiling metaomic data (Lindgreen *et al.* 2016). MerCat2 improves on MerCat v1 (White III *et al.* 2017) with greater parallelization, more considerable scalability, and added visualizations.

Here we describe MerCat2, a tool that can accommodate any size sequence file by utilizing a “divide and conquer” approach that performs integrated analysis, including quality control, *k*-mer counting, and visualization. MerCat2 provides a rapid, robust, versatile analysis of MPS data using DIPA.

## 2 Features

MerCat2 is a versatile and scalable Python-based open-source software package. For massive parallel processing (MPP) and scaling, we developed a byte chunking Algorithm 1 (“Chunker,” see Supplementary Fig. S1) to split files for MPP and utilization in RAY, a massive open-source parallel computing framework to scale python applications and workflows (www.ray.io/). We also implemented a naive *k*-mer counter algorithm in base python for a range of sizes of *k* for versatile counting of both nucleotide and amino acid fasta files (see Supplementary Fig. S2). However, merging large *k*-mer count tables as tabular delimited files (as .tsv) required large memory consumption (>50 GB of RAM). The Dask library we executed in our previous MerCat v1 was responsible for this extensive RAM utilization. To avoid large RAM consumption for streamlining upon low memory systems (e.g. a laptop), we implemented a greedy algorithm that limits RAM usage in native Python even for large datasets (>100 GB of raw sequence data and >60 000 bacterial genomes) (see Supplementary Fig. S3). To plot large principal component analysis (PCAs), which was not a previous feature of MerCat v1, we have utilized and modified an incremental PCA function from sci-kit learn (see Supplementary Fig. S4). MerCat2 scales from laptop to high-performance computing resources, all within the same user-friendly package.

**Figure 1.**
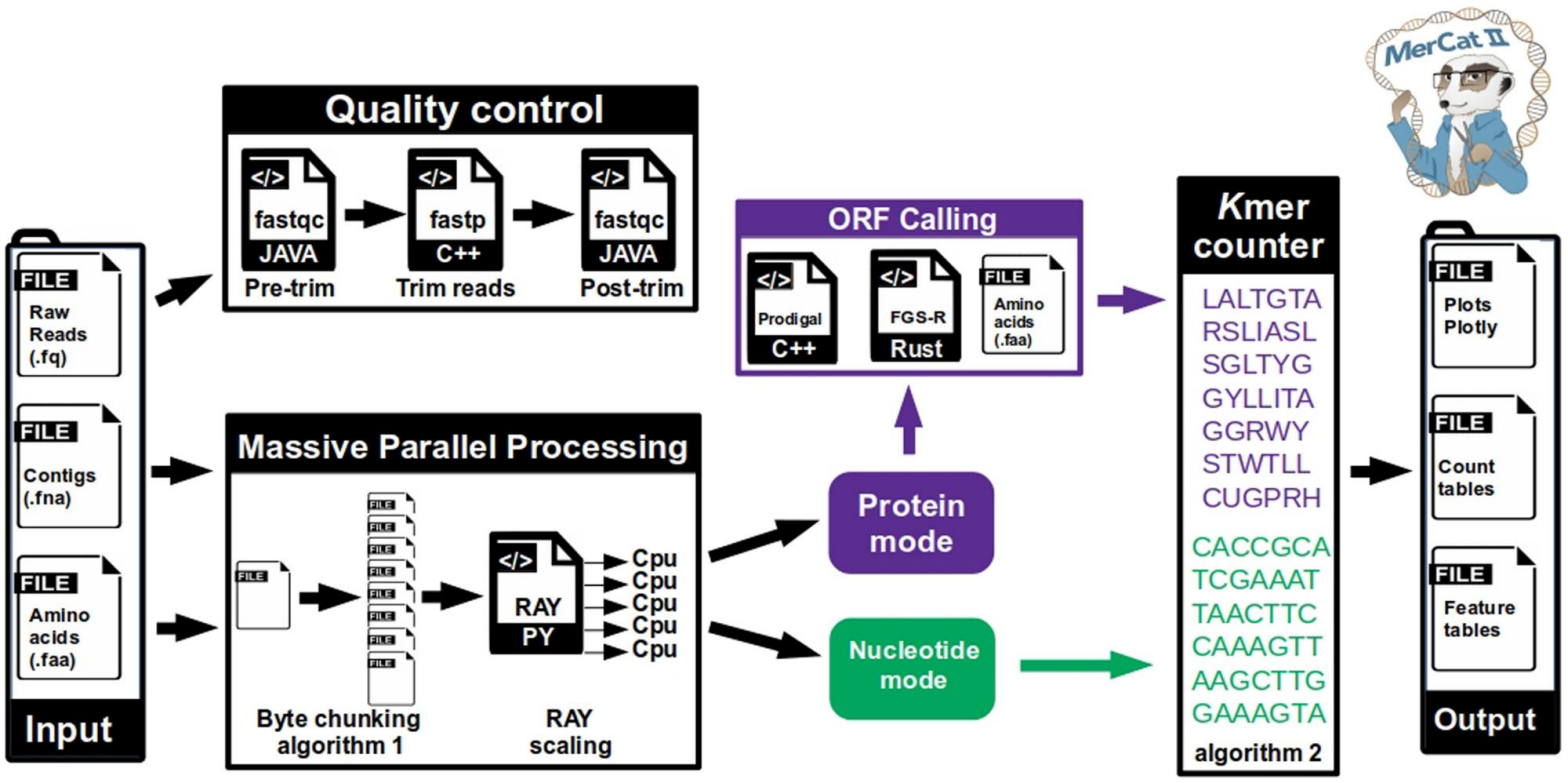
Flow graph of MerCat2. MerCat2 computes *k*-mer frequency counting to any length *k* on assembled contigs as nucleotide fasta, raw reads or trimmed (e.g. fastq), and translated protein-coding ORFs as a protein fasta (i.e. fasta amino acid file,.faa) as inputs. On raw reads, it performs quality control using fastqc (pre/post) trimming with fastp. The MPP occurs with an integration of the byte chunking algorithm 1 (Chunker—Supplementary Fig. S1) with the python library RAY for scaling. MerCat2 can be run in protein or nucleotide mode using the naive *k*-mer counter algorithm 2 (*k*-mer counter—Supplementary Fig. S2). For protein mode, the user can choose Prodigal or FragGeneScanR to convert to ORFs. No ORF calling is required if the user already has an amino acid fasta. Outputs include *k*-mer frequency count tables, protein and nucleotide feature tables, compositional and property dashboard, and PCA ordination (>4 samples).

MerCat2 computes *k*-mer frequency counting to any length k on assembled contigs as nucleotide fasta, raw reads or trimmed (e.g. fastq), and translated protein-coding open reading frames (ORFs) as a protein fasta (i.e. fasta amino acid file, .faa) (Fig. 1). The package also allows for user-defined custom analyses. Although raw read inputs can be used in MerCat2, it is not recommended due to low quality and sequencing errors. Instead, we utilize fastp (Chen *et al.* 2018) for quality control trimming of low-quality data obtained by fastq formats (default trimming is base pair quality score >Q30). For raw reads, MerCat2 provides fastqc reports pre- and post-trimming, which are also included within the final HTML-based report (Andrews 2010) (Fig. 1). Outputs include tabular *k*-mer frequency count tables, tabular ecological diversity metrics (e.g. Alpha diversity), compositional and property dashboard, and PCA ordination (>4 samples). In addition, alpha diversity metrics chao1, ACE, Simpson, Good’s coverage, dominance, and Fisher’s are provided in tabular format (Fig. 1). Outputs also include beta diversity metrics using common dissimilarities/distances such as Bray–Curtis, Jaccard, Canberra similar to Simka and SimkaMin (Benoit *et al.* 2016, 2020). We also offer other dissimilarities/distances such as Euclidean, Cityblock/Manhattan, Chebyshev, Correlation, Cosine, Dice, Hamming, Mahalanobis, Matching, Minkowski, Rogers-Tanimoto, Russell-Rao, Sokal-Michener, Sokal-Sneath, and Yule from python’s scikit-bio library.

MerCat2 has two analysis modes utilizing nucleotide or protein files (Fig. 1). In nucleotide mode, outputs include %G+C and %A+T content, contig assembly statistics, and raw/trim read quality reports are a provided output. For protein mode, nucleotide files (i.e. reads and contigs) can be translated into ORFs using Prodigal (Hyatt *et al.* 2012), which is prokaryotic specific or FragGeneScanRs (Van der Jeugt *et al.* 2022), which works well for general ORF calling. FragGeneScanR provides better gene calling for eukaryotic-rich samples than highly prokaryotic-rich samples. Prodigal is explicitly for prokaryotic gene calling due to the utilization of the Shine-Dalgarno sequence identification (i.e. a ribosomal binding site sequence) that is preferentially found in bacteria, and some archaea but not in eukaryotes (Hyatt *et al.* 2012). Protein property metrics of translated ORFs are provided in tabular format for protein isoelectric point (pI) and hydrophobicity metrics.

## 3 Use cases

To demonstrate the scaling, versatility, and robustness of MerCat2, we compared large microbial genome databases, metagenomic, and metatranscriptomic data from a diversity of samples.

### 3.1 Five genomes example dataset

Our general test dataset includes five bacterial genomes ranging in GC/AT content with high GC >75% *Agrococcus pavilionensis* strain RW1 (White III *et al.* 2013, 2018) and the rest with moderate GC content 50–61% *Azotobacter vinelandii* strain DJ, *Rhizobium leguminosarum*, and two genomes of *Exiguobacterium chiriqhucha* strain RW2 and strain GIC31 that are highly similar (i.e. strains of each other) >97% using average nucleotide identity (White III *et al.* 2019). Example outputs for nucleotide and protein mode are provided in Supplementary Fig. S5.

### 3.2 GTDB archaea/bacterial

We used the GTDB archaea genome database (3412 species) and GTDB bacterial genome (62 291 bacterial species) (Parks *et al.* 2022) to test scalability, disc space, and memory use of *k*-mer counting and PCA plotting. This dataset represents one of the largest high-quality controlled collections of archaea and bacteria genomes. For the GTDB archaea genome database, MerCat2 was able to complete counting whether 4- or 31-mer as either nucleotides or amino acids in under <100 secs, using <15 GB of RAM and <700 Mb of disc space (Table 1). MerCat2 counted GTDB bacterial genome database with whether 4- or 31-mer as either nucleotides or amino acids in <1 h, <30 GB of RAM, and <10 GB of disc space (Table 1).

**Table 1.** Use case data statistics.

Dataset	*k*-mers	Time (seconds)	RAM (GB)	Disk usage
Archaea AA	4	23.08	12.58	699M
Archaea AA	31	28.44	12.97	352K
Archaea NT	4	29.72	11.66	11M
Archaea NT	31	90.92	8.96	32M
Bacteria AA	4	715.87	14.91	20G
Bacteria AA	31	801.59	11.87	841M
Bacteria NT	4	999.36	27.7	389M
Bacteria NT	31	3019.21	27.58	9.9G
Soil AA	4	889.99	32.14	9.4M
Soil AA	31	699.33	31.35	3.1M
Soil NT	4	2663.12	24.2	40K
Soil NT	31	5056.6	19.35	28M
SharkBay AA	4	16.64	18.01	6.6M
SharkBay AA	31	9.43	23.8	32M
SharkBay NT	4	53.49	12.48	36K
SharkBay NT	31	101.02	15.53	228M
Test 5 genomes NT	4	2.07	2.47	8K
Test 5 genomes AA	4	0.96	3.84	1.2M
Test 5 genomes NT	31	5.59	2.48	2.4K
Test 5 genomes AA	31	1.24	3.51	12K

Time in seconds, RAM in Gigabytes, and Disk space used as Kilobytes to MegaBytes. Counting tests uses a minimum *k*-mer count of 10 by default settings.

### 3.3 Shark Bay metatranscriptomes

We included metatranscriptome samples from modern hypersaline microbial mats to test scalability, disc space, and memory for MerCat2. These are 10 samples with five replicate each from smooth and pustular mats from the Nilemah tidal flat located in the southern area of Hamelin Pool, Shark Bay, Western Australia (Campbell *et al.* 2020). The data for all the Shark Bay metatranscriptomes were 15 GB. All 10 samples were counted with either 4- or 31-mer as either nucleotides or amino acids in under 2 min, <20 GB of RAM, and <250 Mb of disc space (Table 1).

### 3.4 Switchgrass soil metagenomes

Eight large soil metagenomes isolated from Lux Arbor, Michigan, were used to test *k*-mer counting and PCA plotting scalability, disc space, and memory use (White III *et al.* 2023). The combined datasets are >5 billion reads, ∼671 million per sample, at >100 GB of data per sample. Scalability for 5 billion metagenomic reads was possible with MerCat2 as it counted all eight datasets with whether 4- or 31-mer as either nucleotides or amino acids in under 90 min, <35 GB of RAM, and <10 Mb of disc space (Table 1).

## 4 Benchmarking

**Figure 2.**
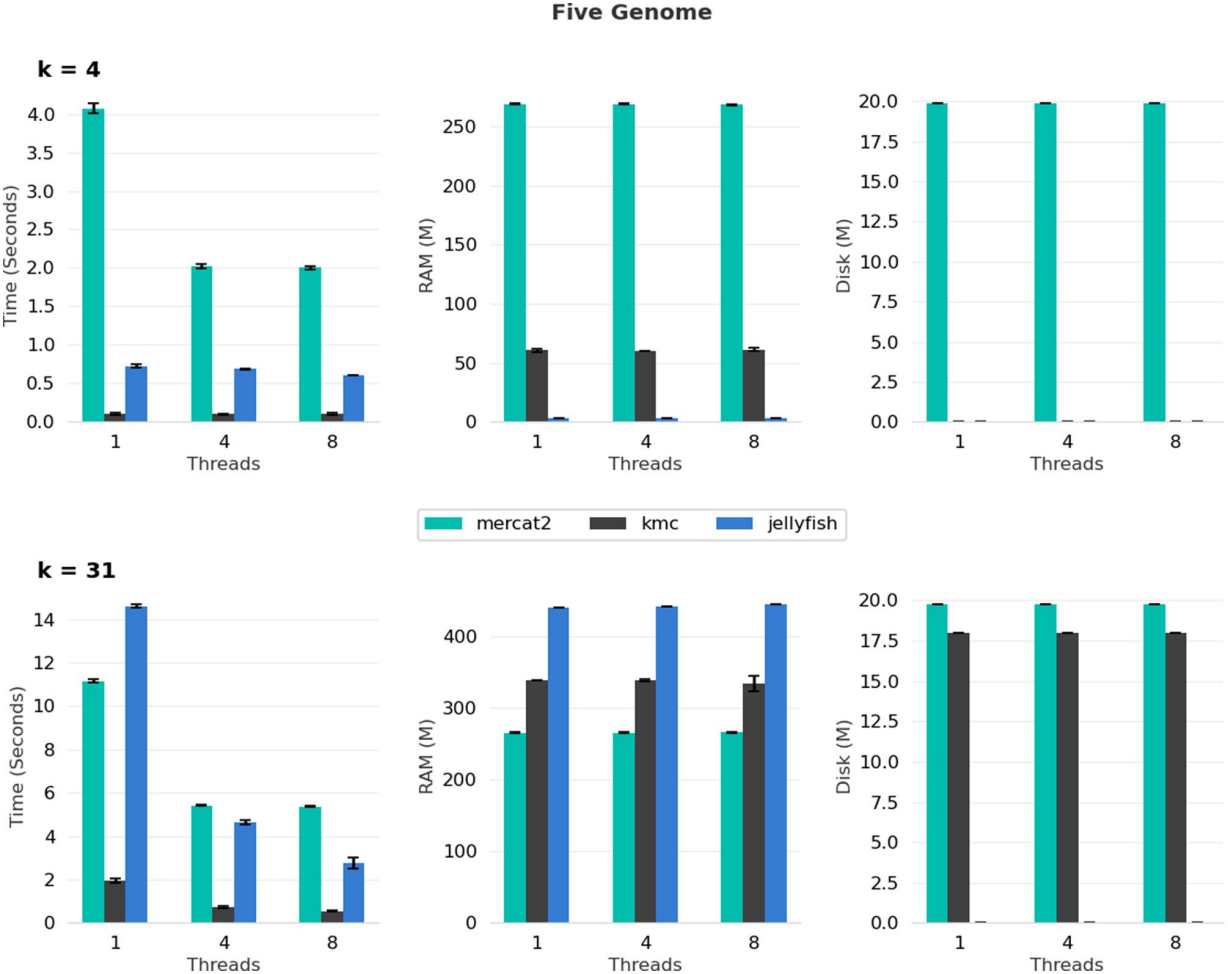
Five genome benchmarking. Five bacterial genomes *Agrococcus pavilionensis* strain RW1, *Azotobacter vinelandii* strain DJ, *Rhizobium leguminosarum*, and two genomes of *Exiguobacterium chiriqhucha* strain RW2 and strain GIC31 were compared against MerCat2, kmc, and Jellyfish using nucleotide whole-genome fasta files for *k* = 4 and *k* = 31. We further compared number of threads from 1, 4, and 8 against all three *k*-mer counters.

**Figure 3.**
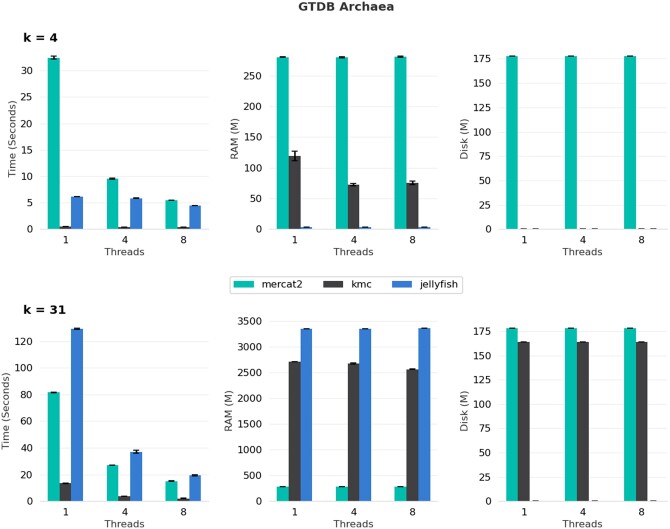
GTDB archaeal genome benchmarking. One hundred randomly selected GTDB archaeal genomes as whole-genome nucleotide fasta where compared MerCat2, kmc, and Jellyfish with *k* = 4 and *k* = 31. We further compared number of threads from 1, 4, and 8 against all three *k*-mer counters.

**Figure 4.**
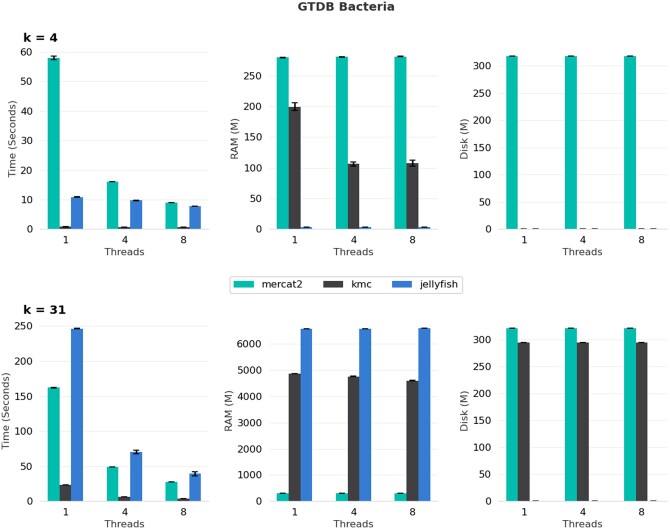
GTDB bacterial genome benchmarking. One hundred randomly selected GTDB bacterial genomes as whole-genome nucleotide fasta where compared MerCat2, kmc, and Jellyfish with *k* = 4 and *k* = 31. We further compared number of threads from 1, 4, and 8 against all three *k*-mer counters.

**Figure 5.**
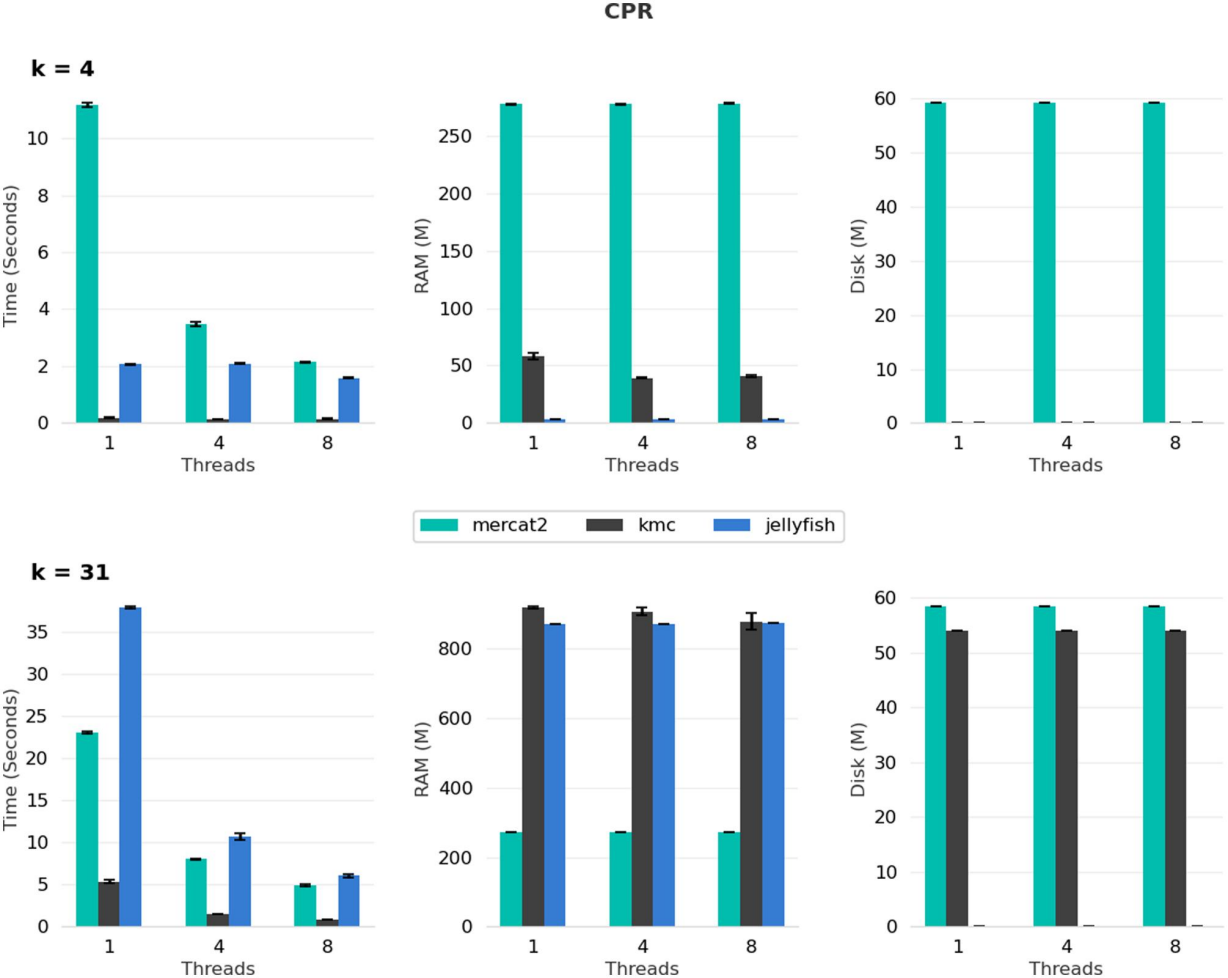
GTDB candidate phyla radiation genome benchmarking. Seventy-eight genomes from the candidate phyla radiation were randomly selected as whole-genome nucleotide fasta where compared MerCat2, kmc, and Jellyfish with *k* = 4 and *k* = 31. We further compared number of threads from 1, 4, and 8 against all three *k*-mer counters.

We benchmarked MerCat2 against two other *k*-mer counters KMC and Jellyfish2 (Marçais and Kingsford 2011, Kokot *et al.* 2017). MerCat2 currently is the only *k*-mer counter that can count both amino acid and nucleotide fasta files; neither are currently supported in KMC or Jellyfish2. For our five-genome benchmark using nucleotide fasta for *k* = 4, MerCat2 is slower, uses more memory, and leaves more disk space; however, with *k* = 31, MerCat2 provides similar speed to Jellyfish2 and utilizes less memory with a maximum of 250 Mb required (Fig. 2). We further benchmarked MerCat2, KMC, and Jellyfish2 against 100 randomly selected GTDB bacteria, GTDB archaea, and 78 genomes from the candidate phyla radiation (CPR). For 4-mer based nucleotide counting single threaded, MerCat2 was the slowest; however, it approached speeds of Jellyfish using single threads, and had a maximum of 250 Mb of RAM required total (Figs 3–5). With 31-mers, MerCat2 was faster than Jellyfish and provided the lowest RAM required (maximum 250 Mb) to complete counting when compared directly to kmc and Jellyfish (Figs 3–5). MerCat2 provides scaling benefits with multiple threads utilization. MerCat2 provides plots and dashboards as html, whereas kmc and Jellyfish do not, thus leaving more disc space post run.

**Figure 6.**
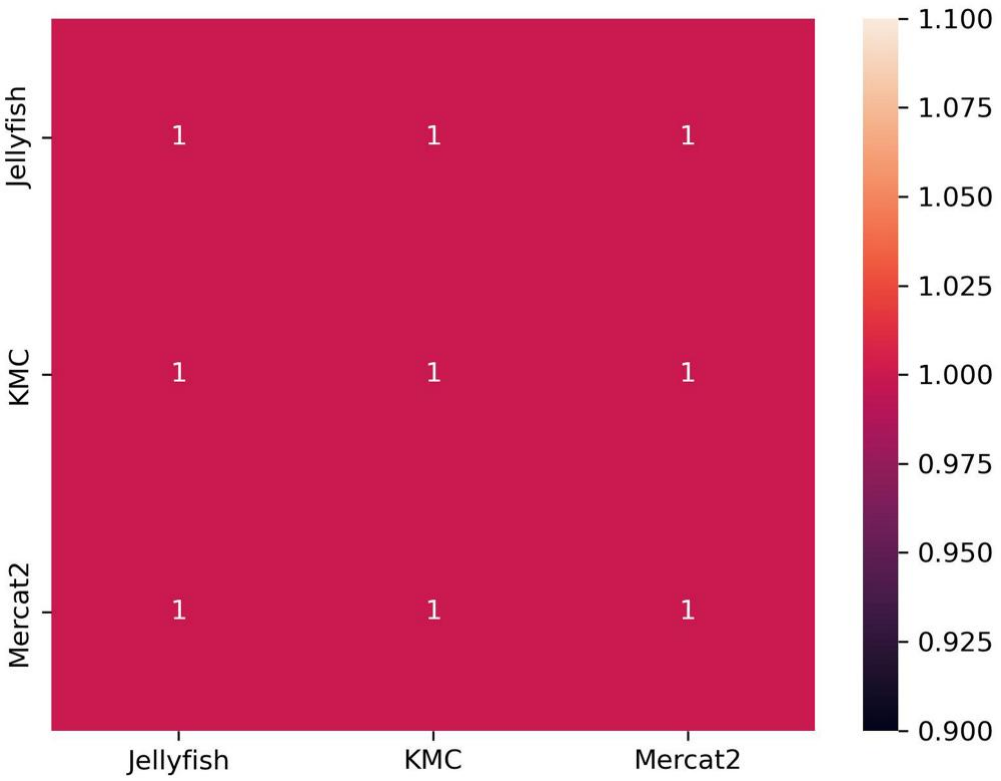
Spearman correlation plot benchmarking. We used our five genomes then counted nucleotide-based *k*-mer using MerCat2, KMC, and JellyFish2 with *k* = 31. We were unable to compare protein (i.e. amino acids) as KMC and JellyFish2 provide no amino acids approaches for inputs.

**Figure 7.**
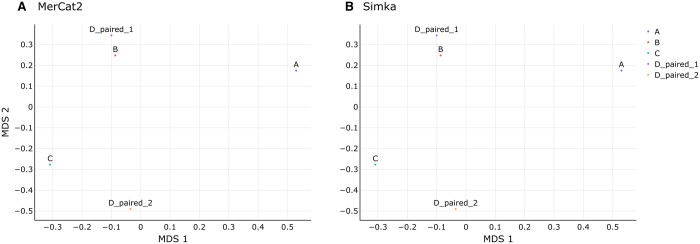
Bray–Curtis dissimilarity calculation comparison MDS. We used the Simka supplied test data for calculation comparison using Bray–Curtis dissimilarity for both (A) MerCat2 and (B) Simka. Both programs used *k* = 21 for *k*-mer enumeration and IDs as suggested by Simka for *k* length.

We directly compared accuracy of *k*-mer identifications (e.g. CCC) and enumeration of those *k*-mers generated by MerCat2 against other commonly used *k*-mer counters such as JellyFish2 and KMC. Using our test five genomes, we counted 31-mers across all three tools (i.e. MerCat2, KMC, JellyFish2) then applied Spearman correlation against the outputs, finding 100% similarity between the outputs (Fig. 6, Supplementary Table S1). We also directly compared MerCat2 and Simka for measuring Bray–Curtis dissimilarity against test data provided by Simka. Bray–Curtis dissimilarity measurement was 100% identical between MerCat2 and Simka (Fig. 7, Supplementary Table S2).

## 5 Conclusions

MerCat2 provides DIPA for metaomic data, utilizing all data present, in a robust, versatile manner resulting in tabular files for downstream analysis, with built-in visualizations and a dashboard. MerCat2 is scalable, accommodating for various input files, user-friendly, easy to install, and user-customizable. MerCat2 provides the same accuracy and precision of *k*-mer identification and enumeration of outputs as JellyFish2 and KMC. MerCat2 is able to calculate both Alpha and Beta diversity metrics on genomes, contigs, amino acids, and reads. MerCat2 when directly compared to Simka provided identical results for the ecological dissimilarity metric (i.e. Bray–Curtis) on the same data. MerCat2 enables rapid analysis of many datasets and large datasets in a database-independent manner.

## Supplementary Material

vbae061_Supplementary_Data

## Data Availability

The data underlying this article are available at github.com/raw-lab/mercat2 and https://osf.io/mzrvj/. The five genomes for benchmarking are available at https://github.com/raw-lab/mercat2/tree/master/data. Shark Bay metatranscriptomes are in OSF under “Shark bay Transcriptomes” at https://osf.io/e94yg/. Lux Arbor soil metagenomes are in OSF under “MMPRNT panicum metagenome mags” at https://osf.io/mzrvj/. The randomly subsampled GTDB bacteria, archaea, and CPR genomes are at https://osf.io/3uz2j/. Simka test dataset is github.com/raw-lab/mercat2/data. For complete GTDB data are available at https://data.gtdb.ecogenomic.org/releases/release207/.
