# Supplementary material for: MerCat2: a versatile k-mer counter and diversity estimator for database-independent property analysis obtained from omics data
Source: Bioinform Adv. 2024 Apr 24;4(1):vbae061. doi: 10.1093/bioadv/vbae061 (PMC11090762; doi:10.1093/bioadv/vbae061)

Combined Nucleotide kmer Summary

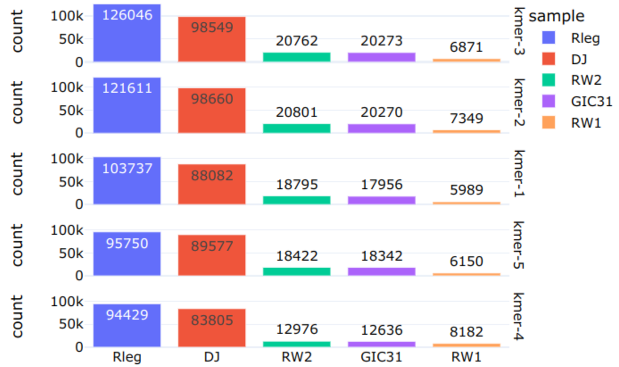

Nucleotide PCA

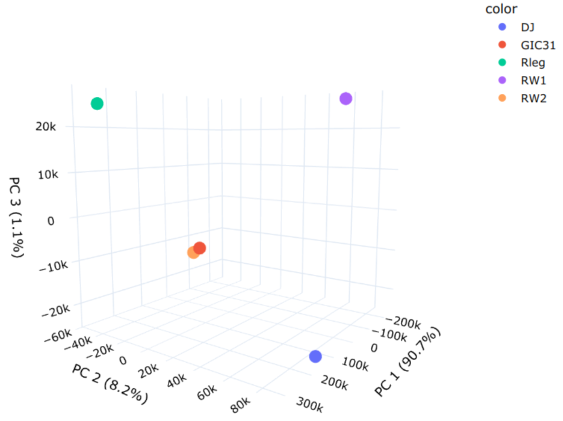

Sample GC Summary

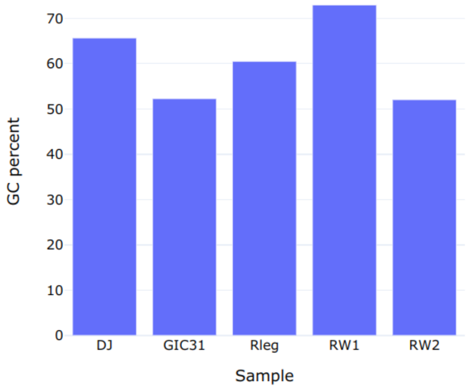

Combined Protein kmer Summary

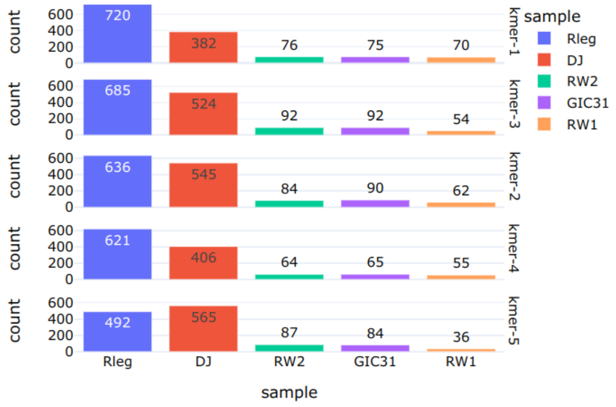

Protein PCA

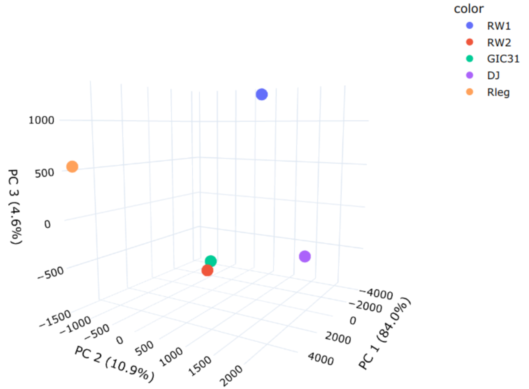

Supplement: vbae061_Supplementary_Data [file vbae061_supplementary_data.zip › Figure_S5.pdf]
