# Supplementary figures and images for: MerCat2: a versatile k-mer counter and diversity estimator for database-independent property analysis obtained from omics data
Source: Bioinform Adv. 2024 Apr 24;4(1):vbae061. doi: 10.1093/bioadv/vbae061 (PMC11090762; doi:10.1093/bioadv/vbae061)

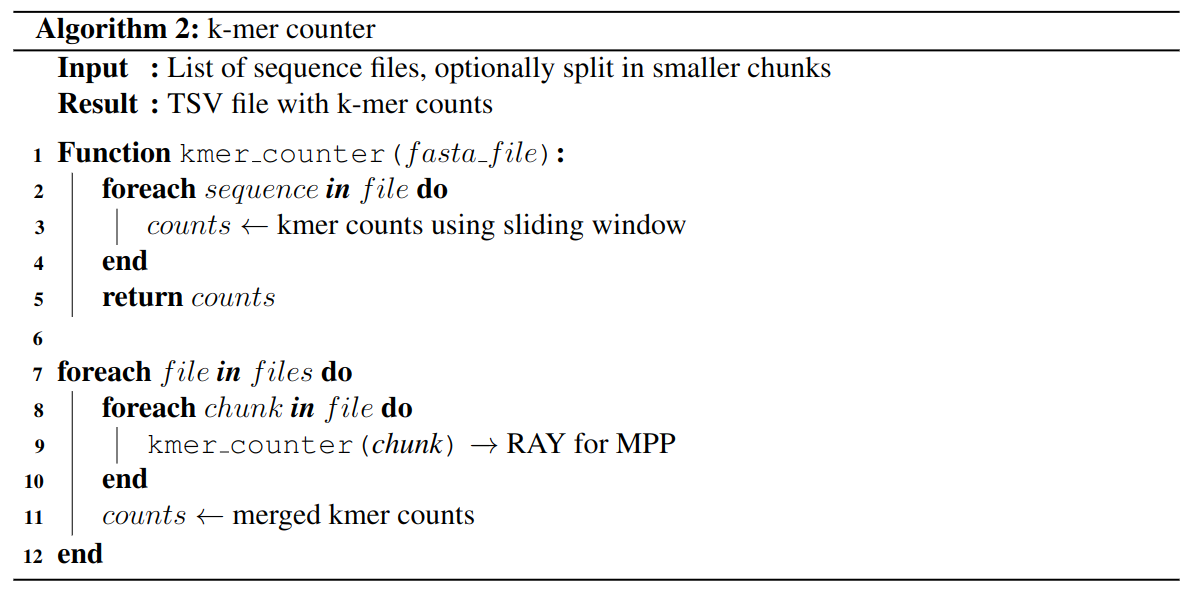

Supplement: vbae061_Supplementary_Data [file vbae061_supplementary_data.zip › Figure_S2.png]

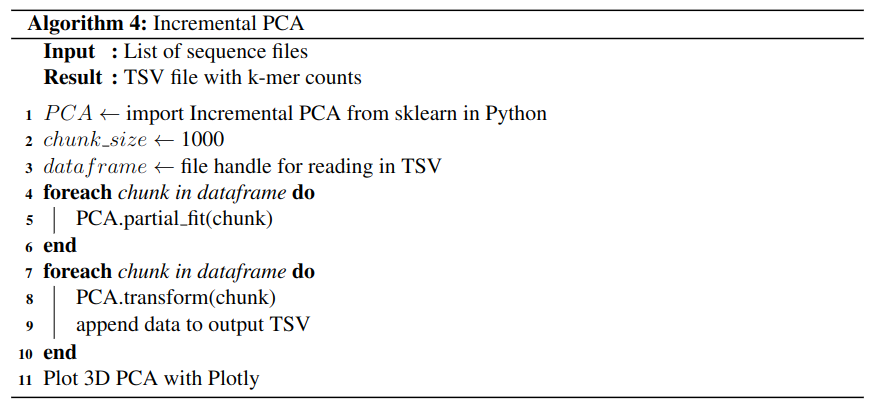

Supplement: vbae061_Supplementary_Data [file vbae061_supplementary_data.zip › Figure_S4.png]

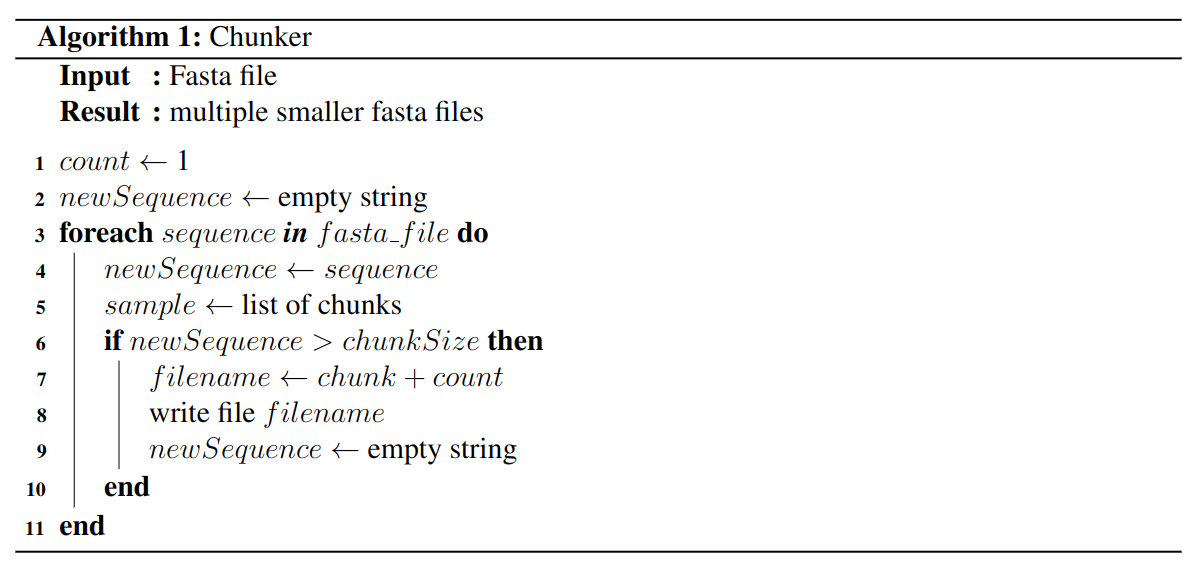

Supplement: vbae061_Supplementary_Data [file vbae061_supplementary_data.zip › Figure_S1.png]

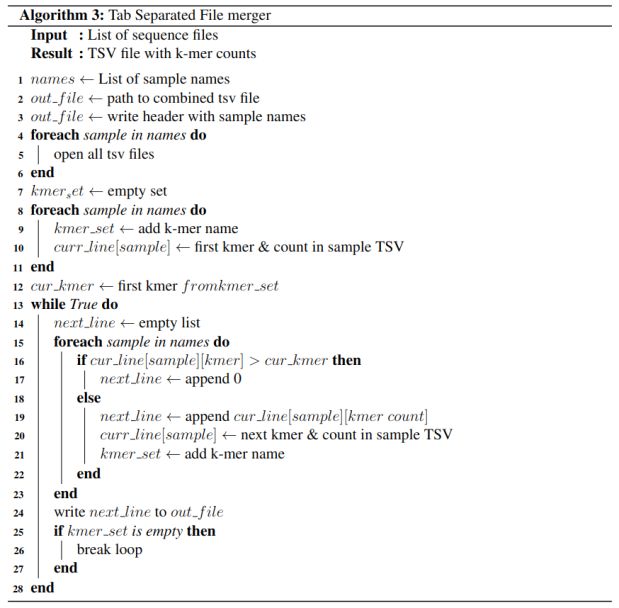

Supplement: vbae061_Supplementary_Data [file vbae061_supplementary_data.zip › Figure_S3.png]
